# Natural Food Supplements Reduce Oxidative Stress in Primary Neurons and in the Mouse Brain, Suggesting Applications in the Prevention of Neurodegenerative Diseases

**DOI:** 10.3390/antiox10010046

**Published:** 2021-01-02

**Authors:** Miriam Bobadilla, Josune García-Sanmartín, Alfredo Martínez

**Affiliations:** Oncology Area, Center for Biomedical Research of La Rioja (CIBIR), 26006 Logroño, Spain; mbobadilla@riojasalud.es (M.B.); jgarcias@riojasalud.es (J.G.-S.)

**Keywords:** oxidative stress, ROS, neurodegenerative diseases, red grape polyphenol extract, white grape seed polyphenol extract, MecobalActive^®^, Olews^®^

## Abstract

Neurodegenerative diseases pose a major health problem for developed countries, and stress has been identified as one of the main risk factors in the development of these disorders. Here, we have examined the protective properties against oxidative stress of several bioactive natural food supplements. We found that MecobalActive^®^, Olews^®^, and red and white grape seed polyphenol extracts may have a neuroprotective effect in vitro, both in the SH-SY 5Y cell line and in hippocampal neuron cultures, mainly by reducing reactive oxygen species levels and decreasing caspase-3 activity. In vivo, we demonstrated that oral administration of the supplements reduces the expression of genes involved in inflammation and oxidation mechanisms, whereas it increments the expression of genes related to protection against oxidative stress. Furthermore, we found that preventive treatment with these natural extracts increases the activity of antioxidant enzymes and prevents lipid peroxidation in the brain of stressed mice. Thus, our results indicate that some natural bioactive supplements may have important protective properties against oxidative stress processes occurring in the brain.

## 1. Introduction

The increasing population lifespan in developed countries is leading to a higher incidence of age-related illnesses, including neurodegenerative diseases (ND) [1]. NDs are characterized by a progressive loss of selectively vulnerable neuron populations in specific brain areas [2]. NDs encompass a heterogeneous group of chronic disorders that include, among others, Alzheimer’s disease (AD) and other dementias, Huntington´s disease, Parkinson´s disease, multiple sclerosis, human prion, and motoneuron diseases [3,4,5,6]. Unfortunately, all these diseases are untreatable at the moment, and, in terms of human suffering and economic and social costs, they represent the fourth cause of global disease burden in developed countries [1]. 

The current literature clearly shows that oxidative stress is one of the main risk factors for AD [7]. The balance between the production of reactive oxygen species (ROS) and reactive nitrogen species (RNS), on the one hand, and of antioxidant substances on the other, is critical for a correct cell function [8]. When unbalanced, the overproduction of ROS and RNS, combined with failing antioxidant defenses, causes oxidative stress [9]. For instance, in AD, a clear diminution of antioxidant activity occurs, which leads to the accumulation of oxidative damage [10]. Additionally, decreased levels of antioxidants such as vitamin C and E and uric acid are observed in AD patients. Many studies have demonstrated that the production of excessive ROS and signs of oxidative stress were detected in the brains of these patients [11,12]. Furthermore, there is evidence that mitochondrial damage resulting in an increased production of ROS contributes to the early stages of the disease prior to the onset of clinical symptoms [9,13]. For these reasons, numerous scientific studies suggest that diets rich in antioxidants may be helpful in preventing, postponing, or controlling the progression of AD [14,15].

To date, there is no effective treatment for these degenerative diseases. Some drugs are used for relieving the symptoms, although they usually generate many side effects and have limited efficacy [16]. Therefore, in order to develop novel preventive therapies, a large number of natural plant extracts have been tested as neuroprotective agents [17]. In nature, there are multiple compounds, including polyphenols, flavonoids, and vitamins, which are capable of counteracting the harmful effects of oxidative stress and reducing the risk of developing NDs [7,18]. Special attention has been paid to flavonoids, a type of polyphenolic compounds that are abundantly present in fruits, vegetables, red and white grapes, and green tea [1]. Flavonoids are nutrients with beneficial health effects derived from their antioxidant and anti-inflammatory properties [19,20]. There is now extensive scientific literature describing the beneficial effects of flavonoids in disease prevention [21,22].

The purpose of the present study was to investigate the protective properties against oxidative stress of several bioactive natural food supplements in vitro and in vivo. The addition of these supplements to commonly used food staples may provide a new and affordable strategy for the prevention of NDs. 

## 2. Materials and Methods

### 2.1. Cell Culture

Human neuroblastoma SH-SY 5Y cell line was obtained from the American Tissue Culture Collection (ATCC, Manassas, VA, USA). Cells were grown in Dulbecco’s Modified Eagle’s Medium (DMEM)-F12 medium (Hyclone, Logan, UT, USA) with 10% fetal bovine serum (Gibco, Carlsbad, CA, USA), 1% penicillin/streptomycin (Gibco), and maintained at 37 °C, 5% CO_2_. Cell culture medium was changed thrice a week.

The cell line was authenticated by STR profiling (IDEXX BioAnalytics, Kornwestheim, Germany).

### 2.2. Primary Hippocampal Neuron Isolation and Culture

Mouse hippocampal neurons were isolated from postnatal day 1 (P1) C57BL/6J mice, as described [23], with slight modifications. Briefly, the hippocampus was dissected in Hank’s balanced salt solution (HBSS) and incubated at 37 °C for 15 min with trypsin/ethylenediaminetetraacetic acid (EDTA) (Sigma-Aldrich, St. Louis, MO, USA). After 3 washes in HBSS, tissue was triturated using a sterile 9-inch Pasteur pipette. HBSS was replaced with Neurobasal plating medium (neurobasal medium, Gibco) containing B27 supplement (1:50) (Gibco), 0.5-mM glutamine solution (Gibco), penicillin/streptomycin (Gibco), 1-mM 4-(2-hydroxyethyl)-1-piperazineethanesulfonic acid (HEPES) (Hyclone), and 10% heat-inactivated donor horse serum (Gibco). Neuroblasts were plated on poly-D-lysine-coated glass coverslips (p96) at a density of 3 × 10^4^ cells/well and placed in a 37 °C, 5% CO_2_ incubator overnight. Next day, in vitro neurobasal plating medium was replaced with neurobasal feeding medium (neurobasal medium containing B27 Supplement (1:50), 0.5-mM glutamine solution, penicillin/streptomycin (1:200), and 1-mM HEPES). Half of the feeding medium was replaced with fresh medium every 4 days.

### 2.3. Natural Extracts

Six commercial natural food supplements were used in this study. They included red grape polyphenol and white grape seed polyphenol extracts (generously provided by Alvinesa Natural Ingredients, Daimiel, Ciudad Real, Spain), extracts from the olive tree (Olews^®^), citicoline, MecobalActive^®^, and Cardiose^®^ (all generously provided by HealthTech Bio Actives, Barcelona, Spain).

Red grape polyphenol and grape seed polyphenol extracts, from Alvinesa Natural Ingredients, are entirely constituted by phenolic compounds (premium selected blending of monomers, dimers, oligomers, and polymers) and have a unique formulation that facilitates direct absorption of the phenolic compounds by the small intestine. All these extracts are currently used as commercial supplements approved for human consumption. Some of these extracts have demonstrated their antioxidant properties in other contexts [24].

### 2.4. Preparation of Aluminum Maltolate

Aluminum maltolate (Al(mal)_3_) was prepared according to published procedures [25]. AlCl_3_·6H_2_O was dissolved in distilled water to a final concentration of 80 mM. Maltolate was dissolved in phosphate-buffered saline (PBS) to a final concentration of 240 mM. The solutions were mixed in equal volumes, and pH was adjusted to 7.4, inducing the precipitation of Al(mal)_3_ crystals. All solutions were filtered using 0.22-μm syringe filters just before use. 

### 2.5. Cell Proliferation Assay

Cell proliferation was analyzed using the Cell Titer 96 Aqueous One Solution Cell Proliferation Assay (Promega, Madison, WI, USA), following the manufacturer’s instructions. Cells were seeded in 96-well plates at a density of 3 × 10^4^ cells per well, allowed to attach for 24 h, and exposed to different concentrations of natural bioactive extracts with or without 125-μM Al(mal)_3_ for 72 h. The MTS reagent (3-(4,5-dimethylthiazol-2-yl)-5-(3- carboxymethoxyphenyl)-2-(4-sulfophenyl)-2H-tetrazolium) was added for 4 h, and absorbance was examined at 490 nm using a microplate reader (POLARstar Omega, BMG Labtech, Ortenberg, Germany). The GI_50_ (growth inhibition of 50% of cells) values of the different compounds were determined using nonlinear regression plots with Prism 8.3.0 (GraphPad Software, San Diego, CA, USA).

### 2.6. Measurement of Intracellular ROS Levels

The levels of ROS were determined in cell cultures by using the cellular ROS assay kit (ab113851, Abcam, Cambridge, UK), following the manufacturer’s instructions. Briefly, SH-SY 5Y cells (8 × 10^3^ cell/well) were incubated with different concentrations of natural bioactive extracts with or without 125-μM Al(mal)3 for 48 h, followed by an incubation with 25-μM 2′-7′dichlorofluorescin diacetate (DCFH-DA) for 45 min at 37 °C in the dark. After two washes with PBS, DCFH-DA was detected by fluorescence spectroscopy, with excitation/emission at 485/535 nm in a microplate reader (POLARstar Omega).

### 2.7. Caspase-3 Activation Assay 

Levels of caspase-3 were determined in cell cultures by using the caspase-3 colorimetric assay kit (K106-100; BioVision Inc., Milpitas, CA, USA), following the manufacturer’s instructions and previous studies [26]. Briefly, enzyme reactions were performed in 96-well microplates, and 50 μL of cell lysate was added to each reaction mixture. Absorbance at 405 nm was measured using a plate reader (POLARstar Omega).

### 2.8. Measurement of Nitrite and Nitrate Concentrations

Cell media were collected and analyzed for their nitrite and nitrate contents by using the nitrite/nitrate colorimetric assay (780001, Cayman Chemicals, Ann Arbor, MI, USA), following the manufacturer’s instructions. NO_X_ (nitrite + nitrate) concentrations were determined by measuring absorbance at 540 nm using a microplate reader (POLARstar Omega). Cell media nitrate concentrations were calculated by subtracting the concentrations of cell media nitrite from the NO_X_ concentrations. 

### 2.9. Restrain Stress and In Vivo Treatments

Six-week-old C57BL/6J mice (Charles-River) were used for this assay. Mice were housed under standard conditions at a temperature of 22 °C (±1 °C) and a 12-h light/dark cycle with free access to food and water. 

Mice were subjected to an acute model of stress by immobilization, as previously described [4,27], by placing them inside 50-mL conical tubes with no access to food or water for the indicated periods of time. Adequate ventilation was provided by several air holes (0.5 cm in diameter) drilled into the conical end of the tube and at its sides. The tubes prevented forward, backward, or rotational movements of the mice. Due to the corticosterone circadian rhythm [28], restraint stress was started at the same time of the day (9:00 a.m.) in all experiments. 

In a pilot study, mice were subjected to restraint for 0, 2, 4, or 6 h, and stress markers were measured (see below). A period of 6h was chosen as the optimal time of restraint for further experiments.

Mice were randomly divided into different experimental groups (n = 8 per group) and received different doses of the natural extracts (or PBS as a control) in 200 µL by oral gavage during 5 consecutive days (Table 1). On the 6th day, mice were subjected to 6 h of restraint stress and immediately sacrificed. The whole brain was dissected out. The olfactory bulbs and the cerebellum were removed, and the remaining tissue was divided into two equal halves by a sagittal section. Each half was frozen separately in liquid N_2_ and stored at −80 °C. One side was used for RNA extraction and the other one for antioxidant enzyme analysis (see below). 

### 2.10. Quantitative Real-Time PCR

Total RNA was isolated from mouse brains and purified as described [34]. Briefly, total RNA was isolated using Trizol reagent (Invitrogen, Carlsbad, CA, USA), with the DNase digestion step performed (Qiagen, Hilden, Germany) according to the manufacturer’s instructions. Resulting RNA (5 µg) was reverse-transcribed using the Superscript III First-Strand Synthesis System for RT-PCR (Invitrogen), and the synthesized cDNA was amplified using SYBR Green PCR Master Mix (Applied Biosystems, Foster City, CA, USA). Transcripts were amplified by real-time PCR (7300 Real-Time PCR System, Applied Biosystems). At the end, a dissociation curve was implemented from 60 to 95 °C to validate the amplicon specificity. For each transcript, a specific calibration curve of cDNA was included to analyze the expression of NADPH oxidase 2 (NOX-2), heme oxygenase (decycling) 1 (HMOX-1), interleukin 6 (IL-6), tumor necrosis factor alpha (TNF-alpha), and nuclear factor erythroid 2-related factor 2 (Nrf-2). All measurements were normalized to glyceraldehyde-3-phosphate dehydrogenase (GAPDH) as a housekeeping gene. Specific primers are shown in Table 2.

### 2.11. Thiobarbituric Acid Reactive Substances (TBARS), Superoxide Dismutase (SOD), and Catalase Activity

For the determination of oxidative stress parameters and antioxidant components in the brain, frozen tissues were homogenized in radioimmunoprecipitation assay (RIPA) buffer (Thermo Scientific, Waltham, MA, USA) supplemented with complete and phospho STOP (Roche, Basel, Switzerland) protease inhibitors. Lipid peroxidation was determined using a commercial TBARS assay kit (CA995, Canvax, Córdoba, Spain). The final malondialdehyde (MDA) products were detected by fluorescence spectroscopy, with excitation/emission at 530/590 nm in a microplate reader (POLARstar Omega). Levels of superoxide dismutase (SOD) activity were determined using an SOD assay kit (CA061, Canvax), according to the manufacturer’s protocols. Absorbance at 450 nm was measured using a POLARstar Omega plate reader. Catalase activities were determined using a commercial catalase activity assay kit (CA063, Canvax) following the manufacturer’s instructions. Enzyme activity was detected by fluorescence spectroscopy, with excitation/emission at 530/590 nm in a microplate reader (POLARstar Omega).

### 2.12. Statistical Analysis

All datasets were analyzed for normalcy and homoscedasticity. Normal data were analyzed by one-way ANOVA and Dunnett’s multiple comparison post-hoc test. Data that did not follow a normal distribution were compared by the Kruskal-Wallis test, followed by the Mann Whitney post-hoc test. Analyses were performed with GraphPad Prism version 8.3.0 (GraphPad Software). A *p*-value < 0.05 was considered statistically significant.

## 3. Results

### 3.1. Olews^®^ and Red and White Grape Extracts Have Neuroprotective Effects on the SH-SY 5Y Cell Line

To test whether the natural extracts used in this study have an antioxidant capacity, in a first approach, we tested them in vitro on the human neuroblastoma cell line SH-SY 5Y.

First, we tested the activity of the chosen supplements (Cardiose^®^, Olews^®^, citicoline, MecobalActive^®^, and red and white grape extracts) on the SH-SY 5Y cell line to study their potential toxicity. The cells were exposed to increasing concentrations of extracts for 72 h, and the cell number was determined by colorimetric methods.

**Figure 1:**
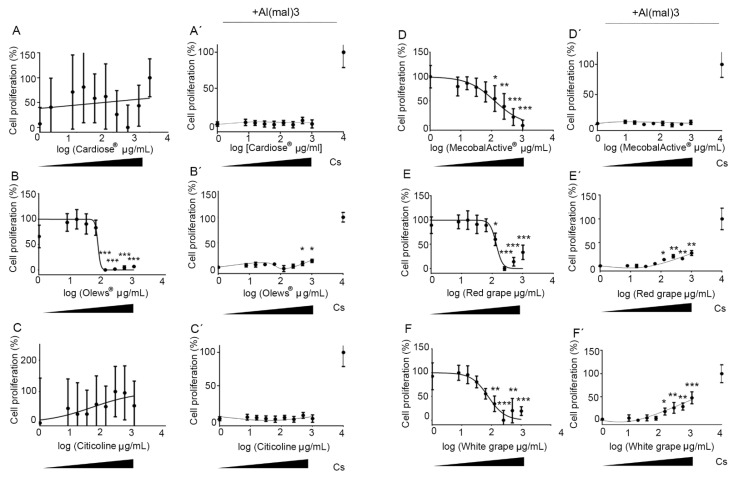
Neuroprotective effects of the extracts on the SH-SY 5Y cell line. Dose-response curve effects of the extracts on the SH-SY 5Y cell line. Cells were incubated with different concentrations of Cardiose^®^ (**A**), Olews^®^ (**B**), citicoline (**C**), MecobalActive^®^ (**D**), red grape (**E**), or white grape extracts (**F**) for 72 h in the absence (**A**–**F**) or presence (**A**′–**F**′) of 125-µM Al(mal)_3_. Data are normalized and expressed as a percentage of the over-basal response (mean ± SEM). Significant differences were analyzed on data from eight different experiments; one-way ANOVA and Dunnett’s multiple comparison post-hoc test were used for statistical analysis. * *p* < 0.05, ** *p* < 0.01, and *** *p* < 0.001 versus cells or Al(mal)_3_ treatment. Cs: control cells, not exposed to Al(mal)_3_.

Interestingly, two different behaviors were observed: (A) extracts that did not elicit significant changes in the number of cells, as with Cardiose^®^ and citicoline (Figure 1A,C), and (B) extracts that induced a dose-dependent toxicity, as observed with MecobalActive^®^, Olews^®^, and red grape and white grape seed extracts. The GI_50_ for these substances were 126, 73, 76, and 134 µg/mL, respectively (Figure 1B,D–F). 

**Figure 2:**
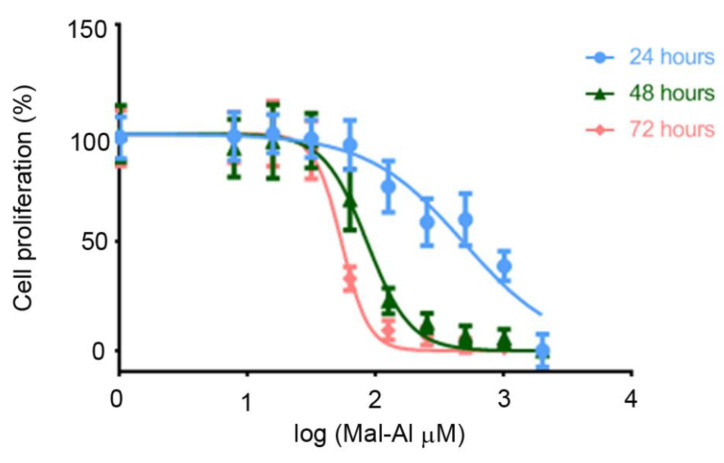
Dose-response curves of the stressor on the SH-SY 5Y cell line. Cells were incubated with different concentrations of Al(mal)_3_ for 24, 48, or 72 h. Data are normalized and expressed as a percentage of the over-basal response (mean ± SEM). Significant differences were analyzed on data from eight different experiments; one-way ANOVA and Dunnett’s multiple comparison post-hoc test were used for statistical analysis; 24 h, *p* < 0.00001; 48 h, *p* < 0.0001; and 72 h, *p* < 0.0001.

Then, we introduced a chemical inducer of cellular stress to assess the neuroprotective effects of the natural extracts. Al(mal)_3_ is a compound that elicits neurotoxicity by inducing mitochondrial membrane potential changes, elevated reactive oxygen species, DNA damage, and apoptosis in SH-SY 5Y cells [35]. Before checking the food supplements, we established the time and concentration curves of Al(mal)_3_ toxicity on the SH-SY 5Y cells. The concentration course studies were carried out at 24 h, 48 h, and 72 h after starting treatment with Al(mal)_3_. We observed that cell death was dose and time-dependent. The GI_50_ concentrations for 24 h, 48 h, and 72 h were 482.60, 85.20, and 53.78 µM, respectively (Figure 2). 

Given these results, we chose 72 h and 125-µM Al(mal)_3_ to perform all in vitro studies involving the stressor. For this, we pretreated the SH-SY 5Y cells with the extracts for 1 h and then exposed them to Al(mal)_3_. After 72 h of incubation, the cell number was assessed. 

In the presence of Al(mal)_3_, Cardiose^®^, citicoline, and MecobalActive^®^ did not significantly improve cell survival (Figure 1A′,C′,D′). On the other hand, Olews^®^ and red and white grape extracts presented a slight recovery of cell proliferation at the highest doses, with GI_50_ values of 47, 930, and 1598 µg/mL, respectively (Figure 1B′,E′,F′). 

### 3.2. Olews^®^, MecobalActive^®^, and Red and White Grape Extracts Have Neuroprotective Effects on Neuroblasts In Vitro

The cytotoxic activity shown for some of the extracts on the tumor cell line led us to ask whether this was specifically an antitumor effect or was due to a broader toxicity. To answer this question, we repeated the experiments using primary cultures of mouse hippocampal neuroblasts. 

**Figure 3:**
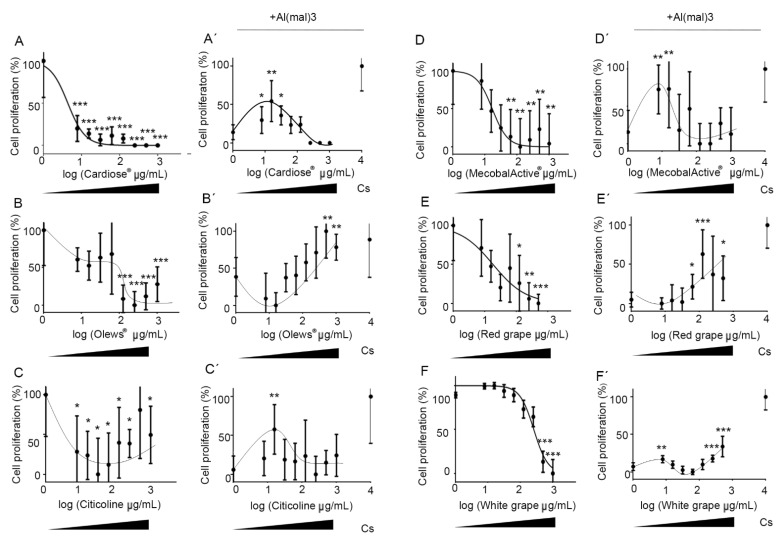
Neuroprotective effects of the extracts on hippocampal neuron cultures. Dose-response curves of the extracts on hippocampal neuron cultures. Cells were incubated with different concentrations of Cardiose^®^ (**A**), Olews^®^ (**B**), citicoline (**C**), MecobalActive^®^ (**D**), red grape (**E**), or white grape extracts (**F**) for 72 h in the absence (**A**–**F**) or presence (**A**′–**F**′) of 125-µM Al(mal)_3_. Data are normalized and expressed as a percentage of the over-basal response (mean ± SEM). Significant differences were analyzed on data from eight different experiments; one-way ANOVA and Dunnett’s multiple comparison post-hoc test were used for statistical analysis. * *p* < 0.05, ** *p* < 0.01, and *** *p* < 0.001 versus cells or Al(mal)_3_ treatment. Cs: control cells, not exposed to Al(mal)_3._

As with the tumor cells, we first tested the activity of the food supplements on hippocampal neuron cultures. As with the SH-SY 5Y cells, we observed a potent and dose-independent toxicity when we added Cardiose^®^ and citicoline to the cell cultures (Figure 3A,C). However, the toxicity was dose-dependent after adding Olews^®^, MecobalActive^®^, and red grape and white grape seed extracts, with EC_50_ values of 16.8, 28.5, 18.2, and 259 µg/mL, respectively (Figure 3B,D–F). 

Next, to study the neuroprotective effects of the natural extracts, we pretreated hippocampal cells with the extracts, and then, we exposed them to Al(mal)_3_. Seventy-two h later, the cell numbers were assessed for all experimental conditions. Olews^®^ and red and white grape extracts presented a slight but significant recovery of the number of cells with the highest doses, with GI_50_ values of 85, 400, and 800 µg/mL, respectively (Figure 3B′,E′,F′). In the case of Cardiose^®^, citicoline, and MecobalActive^®^, there was higher protection by the lower concentrations (7.8 to 15.6 µg/mL) (Figure 3A′,C′,D′). Taken together, these results suggest that Cardiose^®^, Olews^®^, citicoline, MecobalActive^®^, and red and white grape extracts may have certain neuroprotective roles on neuroblasts in vitro.

### 3.3. MecobalActive^®^, Olews^®^, and Red and White Grape Extracts Treatment Reduces ROS Levels and Caspase-3 Activity 

Previous studies found that Al(mal)_3_ induces neurotoxicity in SH-SY 5Y cells by disrupting the levels of ROS and by inducing apoptosis [35]. To find out the mechanisms mediating the neuroprotection role in vitro of Olews^®^, MecobalActive^®^, and red and white grape extracts, we studied both mechanisms in depth. For each extract, we selected a concentration closer to its GI_50_. 

**Figure 4:**
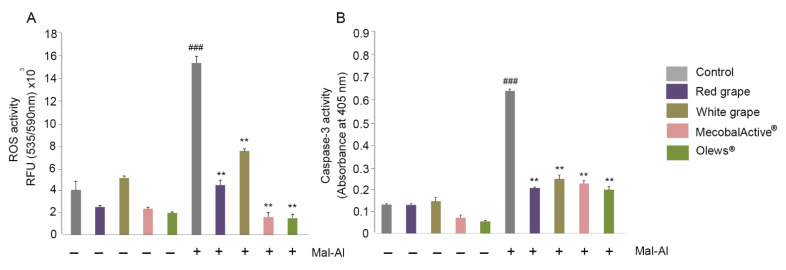
Reactive oxygen species (ROS) levels (**A**) and caspase-3 activity (**B**) on SH-SY 5Y cells after extract treatment. Cells were treated with red grape, white grape, MecobalActive^®^, or Olews^®^ for 48 h in the absence or presence of 125-µM Al(mal)_3_. ROS activity (**A**) was quantified by measuring the fluorescence at 535/590 nm. Caspase-3 activity (B) was quantified by measuring the absorbance at 405 nm. Values are presented as mean ± SEM from at least three independent experiments; Kruskal-Wallis test followed by Mann Whitney post-hoc test were used for statistical analysis. ### *p* < 0.001 versus untreated cells and ** *p* < 0.01 versus Al(mal)3. Abbreviations: Mal-Al: Al(mal)_3._

The ROS measurements indicated that there were no increases in ROS activity elicited by the supplements (Figure 4A). On the other hand, Al(mal)_3_ produced a four-fold increase in ROS activity, as expected (Figure 4A). The ROS levels decreased very significantly when any of the supplements were added in combination with Al(mal)_3_ (Figure 4A). 

In a similar way, the supplements had no effect on the caspase-3 levels of the treated cells, but they greatly and significantly reduced the Al(mal)_3_-induced caspase-3 levels (Figure 4B). No differences were found in the nitrite or nitrate levels (data not shown), indicating that Al(mal)_3_ does not influence the RNS. 

### 3.4. Immobilization for Six h Causes Oxidative Stress in Mouse Brains

Based on our previous findings, we hypothesized that the oral administration of these natural supplements could prevent the appearance of oxidative stress in the brain. Before starting the formal experiments, we investigated which was the shortest period of immobilization needed to cause detectable stress in the mouse brain. For this, the animals were immobilized for zero (control), two, four, or six h, and the mRNA expression of the inflammatory markers IL-6 and TNF-alpha, as well as the oxidation marker NOX-2, were determined in the brain tissue. 

**Figure 5:**
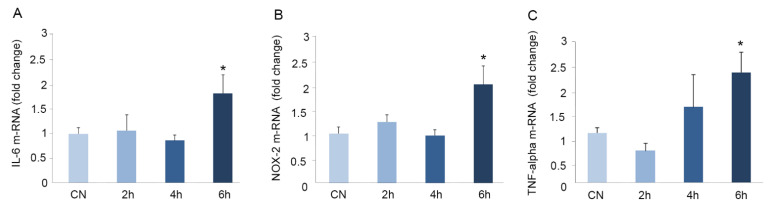
Immobilization causes oxidative stress in mouse brains. Mice were immobilized for different times: 0 (CN), 2, 4, or 6 h. The mRNA expression of IL-6 (**A**), NOX-2 (**B**), and TNF-alpha (**C**) were quantified by real-time (RT)-PCR. The mRNA expression was normalized with GAPDH. All data were related to that from the control and are expressed as a fold change. Values are presented as mean ± SEM from at least three independent experiments. Kruskal-Wallis test followed by Mann Whitney post-hoc test were used for statistical analysis. * *p* < 0.05 versus CN. Abbreviations: CN: control.

We observed a statistically significant increase in the expression of IL-6 (1.7-fold) (Figure 5A), NOX-2 (two-fold) (Figure 5B), and TNF-alpha (2.2-fold) (Figure 5C) only after six h of immobilization. Shorter immobilization times did not result in the significant modification of these markers (Figure 5). For this reason, we chose six h as the optimal immobilization time for further experiments.

### 3.5. Oral Administration of Natural Extracts Provides Protection against Oxidative Stress

**Figure 6:**
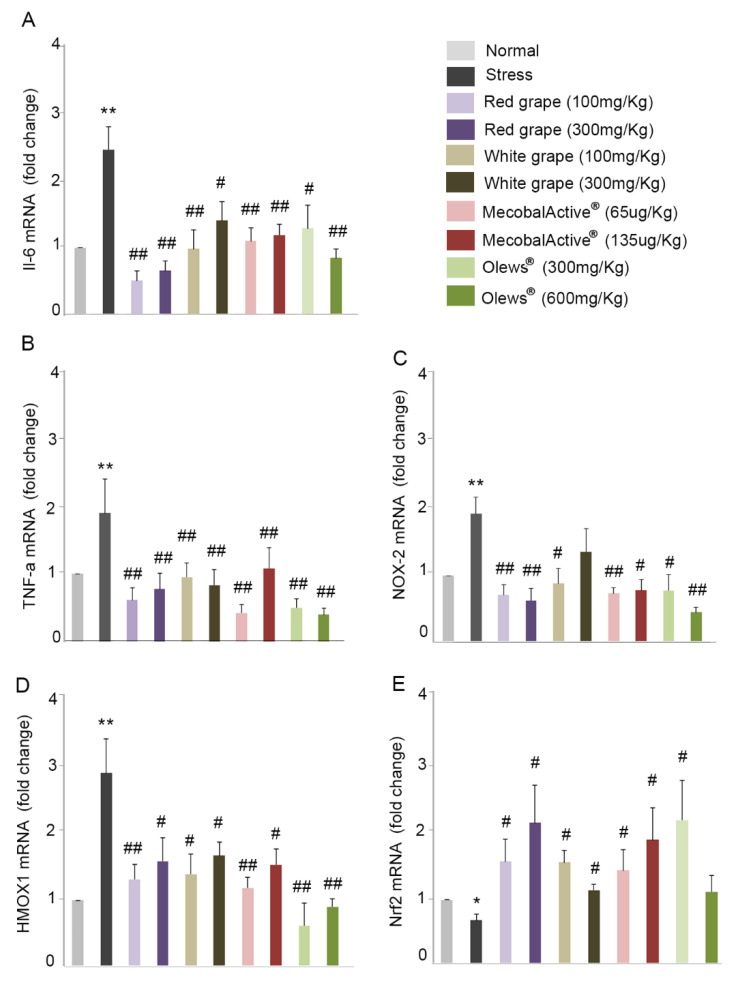
Natural extracts protect against oxidative stress. Red and white grape seed extracts, MecobalActive^®^, and Olews^®^ were administered orally during 5 consecutive days. Then, mice were immobilized for 6 h. The mRNA expressions of IL-6 (**A**), TNF-alpha (**B**), NOX-2 (**C**), HMOX1 (**D**), and Nrf2 (**E**) were quantified in mouse brains by RT-PCR. Gene expression was normalized with GAPDH. All data were normalized to levels found in nonstressed mice (normal) and are expressed as a fold change. Values are presented as mean ± SEM from eight experimental animals. One-way ANOVA and Dunnett’s multiple comparison post-hoc test were used for statistical analysis. * *p* < 0.05 and ** *p* < 0.01 versus normal mice, and # *p* < 0.05 and ## *p* < 0.01 versus restrained mice (stress).

Four natural extracts were selected based on their in vitro behavior and inoculated: red grape, white grape, MecobalActive^®^, and Olews^®^, each of them at two different concentrations (Table 1). In agreement with our previous results (Figure 5), immobilization stress significantly increased the expression of IL-6 and TNF-alpha when compared to the control (2.5-fold and two-fold respectively) (Figure 6A,B). The administration of the extracts resulted in a statistically significant diminution of the expression of both genes in all used conditions (Figure 6A,B). For some of the extracts, specifically red grape, MecobalActive^®^, and Olews^®^, we found values very close to those obtained in the control animals. In addition, we also studied the expressions of NOX-2 and HMOX-1. These genes are involved in oxidation mechanisms, and they increase in the brain of mice subjected to stress [4]. The administration of natural extracts significantly decreased the immobilization-increased expression of both NOX-2 and HMOX-1 (Figure 6C,D). In the same way that occurred with inflammatory cytokines, the extracts brought the expression of both genes to levels very similar to those found in the animals without stress. Finally, we also analyzed Nrf-2 expression. This molecule is a transcription factor that regulates the expression of numerous antioxidant genes. Numerous authors have described Nrf-2 expression as a protective mechanism for oxidative stress [36,37,38]. As expected, immobilization stress reduced Nrf-2 expression (Figure 6E), and all extracts restored Nrf-2 expression to control or even to higher levels, indicating a potent antioxidant effect (Figure 6E). 

### 3.6. Preventive Treatment with Natural Extracts Increases Antioxidant Enzyme Activity in the Brain

To verify the possible protective role of these extracts in oxidative stress, we studied the activity of two antioxidant enzymes, catalase and superoxide dismutase (SOD), in the mouse brains. 

**Figure 7:**
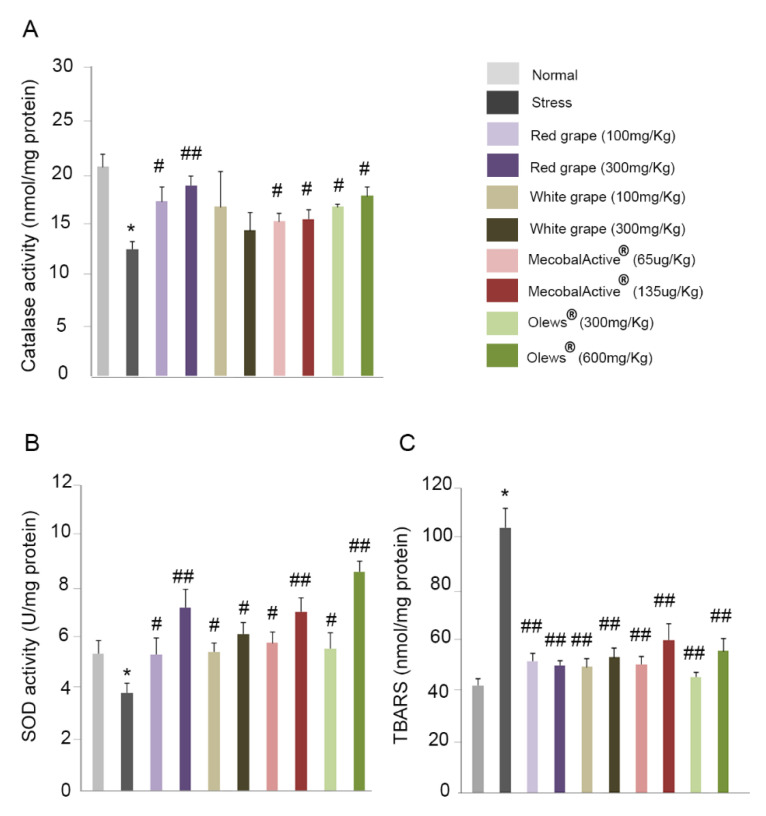
Natural extracts increase the activity of antioxidant enzymes. Mouse brains were isolated, and the catalase activity (**A**), SOD activity (**B**), and TBARS (**C**) were analyzed. The values are presented as mean ± SEM from eight experimental animals. One-way ANOVA and Dunnett’s multiple comparison post-hoc test were used for statistical analysis. * *p* < 0.05 versus normal mice, and # *p* < 0.05 and ## *p* < 0.01 versus restrained (stress) mice. Abbreviations: SOD: superoxide dismutase; TBARS: thiobarbituric acid reactive substances.

It has been described that stress causes a decrease in catalase activity in the mouse brain [4]. First, we confirmed that our experimental model of acute stress was able to reproduce these results. Indeed, we observed a significant reduction in catalase activity in stressed mice compared to nonstressed animals (Figure 7A). Furthermore, the administration of natural extracts led to a statistically significant increase in the levels of catalase activity after the addiction of the red grape extract, MecobalActive^®^, and Olews^®^. No differences were seen after the treatment with white grape extracts (Figure 7A). SOD is one of the most important antioxidant enzymes in cells. It catalyzes the dismutation of the superoxide anion into hydrogen peroxide and molecular oxygen [39]. As with catalase activity, stress caused a significant decrease in SOD activity in the mouse brains (Figure 7B). Interestingly, the administration of natural extracts: red grape, white grape, MecobalActive^®^, and Olews^®^ significantly increased the activity of the SOD enzyme in all used conditions (Figure 7B). 

### 3.7. Treatment with Natural Extracts Prevents the Formation of Lipid Peroxidation Products in the Brain

Lipid peroxidation, an oxidative degradation of cellular lipids, is another important parameter to take into account when studying oxidative stress [40]. We measured the MDA levels present in the mouse brain. Acute stress more than doubled the MDA levels when compared with the nonstressed control group (Figure 7C). In addition, a treatment with any of the extracts drastically reduced MDA levels in the brain tissue, which reached levels very similar to those found in the animals without stress (Figure 7C). 

## 4. Discussion

NDs pose a major health problem for developed countries, and this situation will progressively worsen due to a rapidly ageing population. Stress is known as the “21st century disease” and has been identified as one of the main risk factors in the development of NDs [41]. In this context, the use of natural bioactive extracts has been postulated as a possible preventive treatment of NDs due to their antioxidant power, which is able to reduce stress efficiently [42].

In this work, we found that natural bioactive supplements such as MecobalActive^®^, Olews^®^, and red and white grape seed extracts may have neuroprotective effects in vitro, both in the SH-SY 5Y cell line and in hippocampal neuron cultures, mainly by reducing ROS levels and decreasing caspase-3 activity. In vivo, we demonstrated that oral administration of the supplements for just five days reduces the expression of genes involved in inflammation and oxidation mechanisms, whereas it increments the expression of genes related to protection against oxidative stress. Furthermore, we found that a preventive treatment with these natural extracts increases the activity of antioxidant enzymes and prevents lipid peroxidation in the brains of stressed mice. 

We found that Olews^®^, MecobalActive^®^, and red and white grape seed extracts show a dose-dependent toxicity in SH-SY 5Y cells. Similar results have been described in previous studies. For instance, grape seed extracts were toxic for cell line PC12 at concentrations higher than 200 µg/mL [12]. Similar extracts exhibited a dose-dependent toxicity for oral cancer cell line Ca9-22, which was very significant at doses higher than 100 µg/mL [43]. All these results have been obtained on tumor cell lines, and some authors have proposed that natural antioxidant extracts have an antitumoral capacity [44]. This is why we decided to test the extracts in a primary culture of mouse neurons. To the best of our knowledge, this is the first time that antioxidant extracts were tested in primary cultures, and we were surprised to find that this cellular toxicity also affected the nontransformed cells. Furthermore, with some extracts, the doses needed to elicit a significant antistress response were higher than the GI_50_ value, suggesting that the same treatment was simultaneously cytotoxic and antioxidant. This can be explained if we realize that these extracts are not constituted by a pure substance, but they are a mixture of several chemicals. It is easy to envision a situation in which one or several of the components are cytotoxic, whereas others are antioxidant and, thus, cytoprotective in the presence of a stressor. 

This cytotoxic behavior of the extracts seems to be at odds with the approval of these substances for human consumption and their ample use with no reported side effects. We need to understand that these extracts are approved for oral use (and not as injectable drugs), and therefore, we need to take into consideration the digestive and absorption processes. Digestion could destroy and/or modify some of the extracts´ components, whereas absorption would take only specific substances in such a way that the potentially cytotoxic molecules never reach normal neurons. The vast majority of antioxidant substances need to be fermented by the microbiota of either the small intestine or the colon to achieve optimal absorption [45]. Specifically, Cardiose^®^ contains a flavonoid, diglycoside, that cannot be absorbed in the small intestine. It must proceed to the colon, where it is fermented prior to absorption [46]. Oleuropein, the main component of Olews^®^, is poorly absorbed in vitro [47], although it is fermented by intestinal bacteria, which facilitates intestinal absorption [48]. MecobalActive^®^ needs a carrier protein that serves as a mediator for its intestinal absorption [49]. In the case of grape seed extracts, they need to be digested before reaching circulation [50]. Furthermore, simulated digestion experiments suggest that grape seed extracts are stable in acid-based environments, such as the stomach, but are processed under a simulation of duodenal conditions [51]. Therefore, we have to be cautious when interpreting in vitro results, paying more attention to in vivo studies, which should be more informative about the antioxidant neuroprotector effects of tested supplements. 

Oxidative stress is recognized as a very significant contributor to the pathogenesis of many devastating NDs [52]. In particular, mitochondrial dysfunction leads to the aberrant production of ROS, which are capable of oxidizing lipids and proteins, ultimately causing cell death [53]. We used Al(mal)_3_ to induce neurotoxicity, because it is able to induce mitochondrial membrane potential changes, elevate the ROS, and promote apoptosis in neuron cells [54]. Here, we found that Olews^®^, MecobalActive^®^, and red and white grape extracts reduce Al(mal)_3_–induced ROS in SH-SY 5Y cells. In addition, Al(mal)_3_ causes caspase-3 activation, thus inducing apoptosis and, subsequently, cell death [54]. We also demonstrated that Olews^®^, MecobalActive^®^, and red and white grape extracts were able to reduce Al(mal)_3_-induced caspase-3 activity. In summary, our results suggest that these natural extracts may play certain antioxidant neuroprotective roles in vitro. 

Excessive stress can provoke oxidative stress damage, and the brain tissue has been described as more susceptible to oxidation than other organs [55]. The use of stress models is supported by substantial evidence implicating stress as a precipitating factor for several neuropsychiatric disorders [56]. Most authors in the field use six h of immobilization for their stress-inducing experiments [4,57], but no information of what happens at shorter times is available. We ran a time course and measured the levels of inflammatory cytokines and NOX-2 in brain tissue after two, four, and six h of immobilization. The differences were statistically significant only after the longest exposure (six h), indicating that shorter times do not generate measurable changes in gene expressions in the mouse brain.

Acute restraint stress stimulates several cellular events, resulting in enhanced ROS production [58]. While intracellular ROS serve mainly for host defense against infectious agents, redox-sensitive signal transduction, and other cellular processes, the extracellular release of ROS damages surrounding tissues and triggers inflammatory processes [59] that finally enhance the lipopolysaccharide (LPS)-mediated production of proinflammatory cytokines IL-1β, IL-6, and TNF-α [60,61]. NOX2 is well-known for generating superoxide molecules under oxidative stress-mediated circumstances. Furthermore, HMOX1 acts as a heat shock protein and is induced by oxidative stress [62]. HMOX1 and NOX-2 expressions are upregulated in the stressed brain [63] and in experimental models of NDs [64]. On the contrary, nuclear factor Nrf-2 induces the expression of antioxidant genes that eventually provoke an anti-inflammatory expression profile that is crucial for the initiation of healing [65]. In accordance with this general pathway, we described that the administration of all extracts used in the study (red grape, white grape, MecobalActive^®^, and Olews^®^) prevents the expression of genes involved in inflammation and oxidation mechanisms, while increasing the expression of genes related to protection against oxidative stress, thus identifying them as efficient inhibitors of stress-related cellular damage.

Similarly, restraint stress in rodents precipitates many neurochemical, hormonal, and behavioral abnormalities that are often associated with an imbalance in the brain’s intracellular redox state. Numerous studies have reported that restraint stress enhances lipid peroxidation and decreases antioxidant enzyme activities in rodents [58,66]. To prevent oxidative stress damage, most organisms are equipped with antioxidant mechanisms. SOD and catalase are the best-known antioxidant enzymes [4]. We found that a pretreatment with the extracts increased the activity of catalase and SOD when compared to stressed mice. On the other hand, lipid peroxidation is the oxidative degradation of lipids [67]. MDA is one of the final products of polyunsaturated fatty acid peroxidation in cells. An increase in free radicals causes the overproduction of MDA, which is commonly used as a marker of oxidative stress [68]. In agreement with this, we found that MDA levels significantly increased in the brains of stressed animals but were very efficiently normalized by oral administration of the supplements. 

## 5. Conclusions

Taken together, our results suggest that some natural bioactive supplements (specifically, Olews^®^, MecobalActive^®^, and red and white grape seed extracts) may have important protective properties against oxidative stress processes occurring in the brain. Since oxidative stress has a critical role in the development of NDs, we propose the addition of these natural supplements to commonly used food staples as a possible global preventive treatment for NDs. 

## Figures and Tables

**Table 1:** Food supplements and concentrations used for the in vivo study.

Natural Extract	Dose	References
Red grape	100 mg/kg	[29,30]
	300 mg/kg	
White grape	100 mg/kg	[29,30]
	300 mg/kg	
MecobalActive^®^	65 µg/kg	[31]
	135 µg/kg	
Olews^®^	300 mg/kg	[32,33]
	600 mg/kg	

**Table 2:** Primers used in this study. Annealing temperature was 60 °C for all transcripts.

Gene	Forward Primer (5′-3′)	Reverse Primer (5′-3′)
NOX-2	GCTGGGATCACAGGAATTGT	CTTCCAAACTCTCCGCAGTC
HMOX-1	TGCTCGAATGAACACTCTGG	TAGCAGGCCTCTGACGAAGT
IL-6	ATGGATGCTACCAAACTGGAT	TGAAGGACTCTGGCTTTGTCT
TNF-alpha	CCACCACGCTCTTCTGTCTA	CACTTGGTGGTTTGCTACGA
Nrf-2	AGCGAGCAGGCTATCTCCTA	TCTTGCCTCCAAAGGATGTC
GAPDH	CATGGCCTTCCGTGTTCCTA	GCGGCACGTCAGATCCA

## Data Availability

The data presented in this study are available on request from the corresponding author. The data are not publicly available due to their large volume and little interest.
